# Early recovery trajectories after fast-track primary total hip arthroplasty: the role of patient characteristics

**DOI:** 10.1080/17453674.2018.1519095

**Published:** 2018-10-23

**Authors:** Jarry T Porsius, Nina M C Mathijssen, Lisette C M Klapwijk-Van Heijningen, Jeroen C Van Egmond, Marijke Melles, Stephan B W Vehmeijer

**Affiliations:** 1 Faculty of Industrial Design Engineering, Delft University of Technology, Delft;;; 2 Department of Orthopedics, Reinier de Graaf Groep, Delft;;; 3 Department of Physiotherapy, Reinier de Graaf Groep, Delft, the Netherlands

## Abstract

Background and purpose — Little is known about heterogeneity in early recovery after primary total hip arthroplasty (THA). Therefore, we characterized subgroups of patients according to their hip function trajectory during the first 6 weeks after THA in a fast-track setting.

Patients and methods — 94 patients (median age 65 years [41–82], 56 women) from a single hospital participated in a diary study. Patients recorded their severity of hip problems (Oxford Hip Score, OHS) weekly for 6 weeks after THA. Latent class growth modelling (LCGM) was used to identify patients with the same hip function trajectory and to compare these subgroups on patient characteristics.

Results — LCGM revealed a fast (n = 17), an average (n = 53), and a slow (n = 24) recovery subgroup. Subgroups differed on the estimated weekly growth rate during the first 2 weeks (fast: 9.5; average: 5.3; slow: 2.7), with fewer differences between groups in the last 4 weeks (fast: 0.90; average: 2.0; slow: 1.7). Patients in the slow recovery group could be characterized as women of older age (mean age =69) who rated their health as lower preoperatively, needed more assistance during recovery, and were less satisfied with the outcomes of the surgery.

Interpretation — We identified distinct recovery trajectories in the first 6 weeks after fast-track primary THA which were associated with patient characteristics.

Between 7% and 15% of patients are dissatisfied with the results of total hip arthroplasty (THA) (Espehaug et al. 1998, Adie et al. 2012, Palazzo et al. 2014). Various studies indicate that preoperative patient characteristics, such as age (Dowsey et al. 2010), radiographic osteoarthritis (OA) severity (Keurentjes et al. 2013) and psychological factors (Judge et al. 2011), affect the outcome of THA. It is therefore important to better understand the heterogeneity in responses to THA in order to improve the quality of care for all patients.

Most THA studies investigate postoperative functioning at 6 weeks, 3 months, or 1-year follow-up. However, less is known about differences in hip functioning during the first 6 weeks after discharge. We recently found that patients with a primary THA operated in a fast-track setting improved in self-reported pain and function during the first 6 weeks to clinically meaningful levels and were highly satisfied with the result (Klapwijk et al. 2017). However, a visual inspection of our data also revealed considerable heterogeneity in improvement. A valuable statistical method to improve insight into heterogeneous data is latent class growth modelling (LCGM) (Nagin and Odgers 2010). LCGM has been successfully applied to study responses to treatments in a variety of conditions (e.g. breast cancer [Smoot et al. 2016] and cardiovascular disease [Arthur et al. 2013]).

In the present exploratory study, we aimed to characterize subgroups of patients according to their hip function trajectory in the first 6 weeks after primary THA in a fast-track setting. First, we used LCGM to study whether distinct early recovery patterns after THA can be identified. Second, we investigated whether early recovery patterns are associated with pre- and postoperative patient characteristics.

## Patients and methods

### Study design and setting

This study used data gathered in a recent prospective cohort study, which was conducted at the Reinier de Graaf Hospital (Delft, the Netherlands) (Klapwijk et al. 2017).

Data were gathered with the aim to describe patients’ experiences with THA in a fast-track setting during the first 6 weeks after discharge. Patients were treated in an outpatient setting with the intention of leaving the hospital on the same day as surgery, except when 1 of the following exclusion criteria applied: ASA >2; cardiovascular history; insulin-dependent diabetes mellitus and insufficient support from a caring person at home during the first postoperative night; a preference for an inpatient setting. All patients were operated through the anterior supine intermuscular approach (ASI) by the same orthopedic surgeon.

Discharge criteria for all patients were functional and evaluated by a doctor, a physiotherapist, and a nurse. Patients had to be able to walk 30 meters with crutches or walker, to climb stairs if they were able to walk with crutches, to dress independently, and to go to the toilet independently. Adequate pain relief had to be achieved by means of oral medication before discharge, assessed with a numeric rating scale (NRS, 1–10) for pain below 3 at rest and below 5 during mobilization. Last, the wound had to be dry, or nearly dry, and the patient should not experience dizziness or nausea.

A diary method was used for data collection; patients received a booklet after discharge with a variety of questions regarding their recovery, which they had to complete every day. To reduce patient burden not all questions were assessed daily. Preoperatively (approximately 1 week before surgery) a subset of questions was probed as part of standard routine.

### Participants

Patients who were planned for primary unilateral THA between February 2015 and October 2015 were considered eligible for inclusion. Exclusion criteria were an insufficient command of the Dutch language, being mentally disabled, and a prosthesis in another joint of the ipsilateral or contralateral lower limb placed within 6 months before THA surgery. Of the 144 eligible patients, 43 did not participate in the study (n = 6, co-morbidities; n = 1, insufficient command of Dutch; n = 7, logistical reasons; n = 29, declined participation in the study). Of the 101 included patients, 7 were excluded from analyses for various reasons.

### Outcome measure

The main outcome measure of this study was reported severity of problems with the operated hip, as measured with the Oxford Hip Score (OHS). Higher scores (0–48) indicate better functioning and less pain. Patients had to complete the OHS questionnaire at 1 week before surgery and once every week starting on the 7th day after discharge.

### Patient profiling

We assessed a variety of patient characteristics to explore whether the identified subgroups based on OHS trajectories match the profile of a “typical” patient. These variables can be divided into preoperative or pre-discharge characteristics, healthcare utilization and assistance during the 6-week recovery period, and postoperative patient-reported experience measures (PREMs) after the 6-week recovery period.

### Preoperative or pre-discharge characteristics

Preoperative demographic and patient characteristics included age, sex, BMI, and whether the patient lived alone. Preoperative radiographic OA severity was assessed by the last author (SV) using the Kellgren–Lawrence (K–L) grading (0–4) system. The Charlson comorbidity index (CCI) and the American Society of Anesthesiologists (ASA) physical status classification system (1–4) were used as co-morbidity measures. Health-related quality of life was measured with the EQ-5D (5L). All 5 individual domain scores (1–5) were used, where a higher score indicated more problems, as well as the overall health Visual Analog Scale (0–100; higher score indicated better health). Length of stay (LOS) in hospital was recorded, as well as surgical complications.

### Healthcare utilization and assistance

Once a week patients recorded in their diary whether they had a physiotherapy session, a visit to the doctor, whether they needed aids (e.g., a walking stick or walker) to walk inside or outside their house and whether they received help (e.g., professionally or from a partner) with personal hygiene or with housekeeping. Pain medication was recorded daily.

### Postoperative PREMs

At the end of the 6-week recovery period patients completed the PREMs in their diary. They indicated on an 11-point scale how satisfied they were with the results of surgery (0–10). On several 5-point scales they indicated their satisfaction with the general result of surgery, current pain, activities of daily living, and their quality of life. In addition, we asked whether they would opt for THA surgery again if needed (yes/no) and if they would recommend it to others (yes/no). Patient’s attitude towards fast-track THA, provided support during recovery, and the information provided were also assessed through yes/no questions.

### Statistics

To investigate heterogeneity of patients’ responses to THA, we conducted LCGM analyses using a group-based trajectory modelling approach (Nagin and Odgers 2010) on the OHS assessed at 7 time-points. Robust full-information maximum likelihood estimation (Mplus version 6.12; https://www.statmodel.com/version6.12.shtml) was used to handle missing data. LCGM is an extension to latent growth curve models, which are commonly used to assess inter-individual differences in intra-individual change (Duncan and Duncan 2004). In standard latent growth curve models 1 intercept (initial value) and 1 slope (change) is estimated to describe overall growth in a sample. In contrast, LCGM allows an investigator to estimate separate intercepts and slopes for a number of unobserved classes (i.e., subgroups). The goal of LCGM analyses is to identify different clusters in such a way that homogeneity is maximized within classes and heterogeneity between classes (Hoeksma and Kelderman 2006).

Previous analyses on the same data showed a non-linear improvement in the OHS with a different rate of change from week 2 onwards (Klapwijk et al. 2017). We used a piecewise growth model specification (Chou et al. 2004) to accommodate for the non-linear growth. In such a model a growth process is cut into 2 (or more) pieces representing the average growth rate in a specific time period. In our case the 1st slope represents the average weekly growth rate between baseline and week 2, and the 2nd slope represents the average weekly growth rate between week 2 and 6. In line with the group-based trajectory modelling approach, no random effects were estimated.

We compared 1- to 4-class models and used conventional criteria to assess their relative statistical fit (BIC, BLRT, Entropy) (Nylund et al. 2007). Based on statistical fit, as well as theoretical and clinical interpretability of these models, 1 model was chosen as a basis to explore patient profiles. The most likely class membership based on posterior probabilities of the chosen model was used to assign every patient to the appropriate class. To compare classes, averages or percentages were calculated for all patient profiling characteristics. To assist the profiling process, we ran multinomial logistic regression analyses with class membership as outcome, and the specific characteristic in question (e.g., age) as predictor. As reference class the largest class will be chosen. Statistically significant differences (p < 0.05) should be interpreted as exploratory results and are displayed for descriptive purposes.

### Ethics, funding, and potential conflicts of interest

The study protocol was assessed by the regional Medical Ethical Committee and no ethical approval was necessary, as the study did not fall under the scope of the Medical Research Involving Human Subjects Act. However, all included patients gave their written informed consent. We thank the Foundation for Scientific Research of Reinier de Graaf Groep, Delft, for funding this study. SV has a consultant contract with Zimmer Biomet. The authors have no further competing interests to declare.

## Results

### Patient characteristics

Patients were median 65 years (41–82) old and 56 were women (Table 1). Almost all patients were classified as ASA I or II. Of all 94 patients 80 had no missing values on the OHS, 13 had a missing value on 1 occasion, and 1 on 2 occasions.

**Table 1.** Pre- and postoperative characteristics of patients according to class membership

	Class 1:	Class 2:	Class 3:	
	Fast	Average	Slow	Total
	recovery (n = 17)	recovery (n = 53)	recovery (n = 24)	recovery (n = 94)
Preoperative or pre-discharge characteristics				
Female sex, n	8	29	19^a^	56
Age, mean (SD)	59 (8.6)^a^	65 (8.3)	69 (7.3)^a^	65 (8.7)
BMI, mean (SD)	27 (5.3)	27 (3.5)	28 (4.2)	27 (4.1)
ASA score, n				
1	9	18	7	34
2	8	32	15	55
3	0	3	2	5
Charlson comorbidity index, n				
0	11	28	9	48
1	5	15	7	27
≥ 2	1	10	8	19
Kellgren–Lawrence classification >3, n	12	41	16	69
Outpatient surgery, n	13^a^	25	4^a^	42
Length of stay in nights, median (IQR)	0.0 (0.5)^a^	1.0 (1.0)	1.0 (1.0)^b^	1.0 (1.0)
Operated side, left, n	12^a^	20	12	44
Discharged to home, n	17	52	22	91
Living alone, n	1	6	5	12
Additional prostheses, n	3	6	9^a^	18
No surgical complications, n	17	51	22	90
Oxford Hip Score, mean (SD)	24 (7.5)	24 (8.5)	23 (9.4)	24 (8.5)
EQ–5D, median (IQR)				
mobility (1–5)	2.0 (0.0)	2.0 (0.0)	2.0 (0.0)	2.0 (0.0)
self-care (1–5)	1.0 (1.0)	1.0 (1.0)	1.0 (1.0)	1.0 (1.0)
usual activities (1–5)	2.0 (1.0)	2.0 (0.0)	2.0 (0.0)	2.0 (0.0)
pain/discomfort (1–5)	2.0 (0.0)	2.0 (1.0)	2.0 (1.0)	2.0 (1.0)
mental health (1–5))	1.0 (0.0)	1.0 (0.0)	1.0 (0.0)	1.0 (0.0)
VAS (0–100), mean (SD)	70 (18)	71 (17)	61 (26)^a^	69 (20)
NRS pain in rest (0–10), mean (SD)	4.4 (2.6)	4.6 (2.4)	6.0 (2.5)^a^	4.9 (2.5)
NRS pain movement-evoked (0–10), mean (SD)	6.5 (2.0)	7.0 (1.9)	7.2 (1.9)	7.0 (1.9)
Healthcare utilization and assistance during recovery				
Number of weeks, median (IQR)				
with attended physical therapy sessions	5.0 (1.0)	6.0 (1.0)	6.0 (1.0)	6.0 (1.0)
with a doctor’s visit	0	0	0	0
with pain medication usage	3.0 (2.0)^b^	5.0 (3.0)	6.0 (1.0)^a^	5.0 (3.0)
with personal hygiene assistance	2.0 (2.0)^a^	3.0 (4.0)	6.0 (2.8)^a^	3.5 (4.0)
with housekeeping assistance	5.0 (3.0)	6.0 (1.0)	6.0 (1.0)	6.0 (1.0)
when walking aids were required inside	3.0 (2.5)^b^	5.0 (2.0)	6.0 (1.0)	5.0 (3.0)
when walking aids were required outside	2.0 (2.5)^b^	4.0 (2.0)	5.0 (1.0)^a^	4.0 (2.0)
Postoperative PREMs^c^				
Satisfaction, median (IQR)				
with result THA (0–10)	10 (0.0)	10 (1.0)	8.0 (3.0)^b^	10 (2.0)
with general result THA (1–5)	5.0 (0.5)	5.0 (1.0)	4.0 (0.0)^b^	5.0 (1.0)
with current pain (1–5)	5.0 (0.5)	4.0 (1.0)	4.0 (2.0)^b^	4.0 (1.0)
with activities of daily living (1–5)	5.0 (1.0)	4.0 (0.0)	4.0 (1.0)^b^	4.0 (1.0)
with quality of life (1–5)	5.0 (1.0)	4.0 (1.0)	3.5 (1.0)^b^	4.0 (1.0)
Would have THA again if needed, n	17	50	20	87
Would recommend to others, n	17	50	21	88
Positive attitude fast-track THA, n	15	47	18	80
Positive attitude assistance during recovery, n	16	48	18^a^	82
Positive attitude provided information, n	9	40	17	66

a p < 0.05, compared with the average recovery class 2.

b p < 0.01, compared with the average recovery class 2.

c PREMs: Patient reported experience measures

### Early recovery trajectories

As can be seen from the BIC values and the outcomes of the BLRT, model fit improved by adding classes up to the 4-class model (Table 2, see Supplementary data). The entropy values all exceeded the minimum of 0.8, indicating that the classes were sufficiently distinct from each other.

**Table 2.** Piecewise latent class growth model results

Number of classes Intercept^a^ (SE) Slopes^b^ (SE) free parameters		BIC^c^		BLRT^d^		Number of Entropy^e^ Patients per class			
1-class	Class 1	24 (0.76)	5.41 (0.47)	10	4291.627	–	–	94	
2-class	Class 1	22 (1.2)	3.94 (0.61)	21	4024.293	p < 0.001	0.92	49/45	
	Class 2	25 (1.0)	7.09 (0.74)						
3-class	Class 1	25 (1.9)	9.52 (0.91)	32	3912.545	p < 0.001	0.96	24/17/53	0.90 (0.12)
	Class 2	24 (0.89)	5.30 (0.46)	2.03 (0.15)					
	Class 3	22 (1.6)	2.68 (0.89)	1.73 (0.39)					
4-class	Class 1	25 (0.96)	5.84 (0.51)	43	3867.885	p < 0.001	0.94	26/33/18/17	1.93 (0.20)
	Class 2	24 (1.3)	4.54 (0.75)	1.86 (0.21)					
	Class 3	20 (1.7)	2.57 (0.84)	1.92 (0.52)					
	Class 4	25 (1.9)	9.56 (0.94)	0.89 (0.13)					

a Estimated average OHS at preoperative baseline.

b The first number refers to the estimated weekly growth rate in the first 2 weeks of the 6-week recovery period; the second number refers to the estimated weekly growth rate in the last 4 weeks of the 6-week recovery period.

c Bayesian Information Criterion, a lower value indicates better model fit.

d Bootstrapped Likelihood Ratio Test, tests whether a significant improvement in model fit occurred with the addition of an extra class.

e Entropy index (0–1), higher values indicate better overall accuracy of class separation.

When accounting for the fit of the various models, as well as the clinical interpretation based on the intercept and slopes, we preferred the 3-class model. The 2-class model only distinguishes between an average and faster initial growth group and does not identify a—theoretically expected—slower recovery group. In the 3-class model this latter group is identified (Class 3), as well as an average (Class 2) and fast recovery group (Class 1), and statistical fit improved. Even though fit improved even further in a 4-class model, clinical interpretability did not improve. In this model the average recovery subgroup in the 3-class model was divided into 2 separate subgroups (Class 1 and 2) differing only marginally from each other in their initial growth rate. We therefore chose the 3-class model for further analyses.

The average change in OHS from baseline until week 6 for the fast recovery group was 23, for the average recovery group 19, and for the slow recovery group 9.9 (Figure). The biggest difference between the classes became apparent in the first 2 weeks of recovery. Both the fast (Class 1) and average recovery group (Class 2) showed a steep initial increase in reported functioning of the operated hip in the first 2 weeks, levelling off in the subsequent 4 weeks. The levelling off phase was a little more pronounced in the fast recovery group. In stark contrast, the slow recovery group showed a slow and steady improvement, lacking the exponential increase in the first 2 weeks. In addition, growth seemed to already level off after week 4. It is also notable that the classes were similar in their preoperative baseline OHS.

### Differences between classes in patient characteristics

There were distinct differences between the 3 different early recovery trajectory classes in preoperative characteristics, healthcare utilization and assistance during recovery, and postoperative PREMs.

### Average recovery patient profile

The largest class of patients (Class 2, average recovery) can be characterized as slightly overweight patients (average BMI =27) with an average age of 65, having an average preoperative movement-evoked NRS pain score of 7 and staying on average 1 night in hospital after surgery. Patients in this class on average did not report mental health problems preoperatively or problems with self-care, but they did report slight problems with mobility, usual activities, and pain. For the majority of the 6-week recovery period they used walking aids, attended weekly physical therapy sessions, and took pain medication. After 6 weeks they were highly satisfied with the results of the surgery. In the subsequent analyses we compared the other smaller classes with this dominant class.

### Fast recovery patient profile

The patients in Class 1 (fast recovery) were younger (mean age =59) and were operated more often in an outpatient setting. During the 6-week recovery they used pain medication and required walking aids for a shorter period (median =3 weeks) than patients in the average trajectory class (median =5 weeks).

### Slow recovery patient profile

Patients in Class 3 (slow recovery) were more often female (four-fifths) and older (mean age =69) than patients classified in the average recovery trajectory. They were less often operated on in an outpatient setting and rated their overall health as lower and reported more hip pain at rest before the surgery. More patients already had another prosthesis, and although not statistically significant, co-morbidities (ASA and CCI) were more common. Compared with the average recovery class patients required more weeks with personal hygiene assistance (median =6 weeks) and longer use of walking aids outside (median =5 weeks). After the 6-week recovery period patients were less satisfied with the results of the surgery and were also less positive about the support provided during recovery.

## Discussion

In this prospective cohort study we identified 3 different trajectories of early recovery after primary unilateral THA in a fast-track setting.

In the slow recovery group, healthcare utilization and assistance during recovery was higher, in combination with lower satisfaction of provided care. Preoperatively, health perception was more negative and patients reported more pain at rest. Together with being older and having more co-morbidities, clinicians might describe this group of patients as being more “difficult” (Hahn et al. 1996, Jackson and Kroenke 1999) or challenging. The more complex preoperative clinical picture may have played a role in the slower recovery after THA. Previous work has for instance shown that preoperative pain at rest (as opposed to movement-evoked pain) is associated with chronic pain after THA (Sayers et al. 2016). Pain at rest could be an indication of altered central pain modulation (Baert et al. 2016), which might have played a role in this patient subgroup.

At the other end of the spectrum the subgroup of patients displaying a fast recovery presented a very different profile. The majority of these patients left hospital on the day of surgery. In line with their pronounced improvement in pain and function during the first 2 weeks after discharge, they used pain medication and walking aids for a much shorter period of time than the average patient subgroup. Their quick recovery may in part be explained by having fewer co-morbidities and their younger age. However, these patients did not rate their health as better, or have less pain preoperatively. Perhaps being scheduled for the outpatient treatment and meeting the discharge criteria to leave on the same day as the surgery may have instilled more positive recovery expectations. Positive outcome expectations can enhance the effects of medical treatments (Crow et al. 1999) and may have led to the quicker recovery we observed in this subgroup of patients.

Our results underscore the need for tailored care and provide directions to achieve such an outcome. Patients fitting the slow recovery trajectory were less satisfied with the surgery and less positive about the support provided during recovery. Although we are not able to tell what kind of support this subgroup of patients wanted, their higher healthcare utilization suggests unfulfilled needs when it comes to pain management and assistance with daily activities. This is in line with previous work stressing the importance of postoperative pain management after THA to improve patient satisfaction (Bergés et al. 2006). It may be of value to investigate this subgroup’s needs for care management and cost-effective ways for improvement.

The most important limitations concern the generalizability of our findings and that we did not try to identify potentially important preoperative psychosocial predictors of early recovery. Being an exploratory study with a relatively small sample size, replication of our findings in other larger samples in different settings is essential.

Previous studies have pointed to the importance of psychosocial predictors of responses to arthroplasty, such as pain catastrophizing or psychological distress (Vissers et al. 2012, Lewis et al. 2015). A recent controlled cohort study demonstrated a positive effect of providing psychological support on well-being and physiotherapy objectives after THA (Tristaino et al. 2016). When looking at the broader treatment spectrum for OA, a recent meta-analysis calculated that 75% of all treatment effects were due to contextual effects (i.e., placebo effects) (Zou et al. 2016). This is in line with studies showing that patients with higher preoperative pain relief expectations showed better outcomes after arthroplasty (Gandhi et al. 2009, Judge et al. 2011). The only preoperative psychosocial predictor in our study was self-rated mental health, measured with 1 question from the EQ-5D questionnaire. We did not find a difference between the groups; however, a more comprehensive measurement of psychosocial factors might have revealed more differences between the recovery groups.

In summary, 3 distinct subgroups of patients were identified with markedly different recovery rates. By comparing these groups we were able to develop preliminary patient profiles of early recovery. Our study demonstrates the necessity to further explore the specific needs of these different subgroups, as well as clinical and psychosocial predictors of early recovery.

### Supplementary data

Table 2 is available available as supplementary data in the online version of this article, http://dx.doi.org/ 10.1080/17453674. 2018.1519095

The authors are grateful to all the patients for their efforts in completing their diaries, and especially thank N. de Esch for her help during the study.

JP performed data analysis, wrote and revised the manuscript, NM designed the study, supported data analysis and reviewed the manuscript. LK supported data collection and reviewed the manuscript. JE helped composing the diary, performed data collection, and reviewed the manuscript. MM supported the data analysis and reviewed the manuscript. SV designed the study, operated on all patients and reviewed the manuscript.

*Acta* thanks Henrik Husted for help with peer review of this study.

**Figure:**
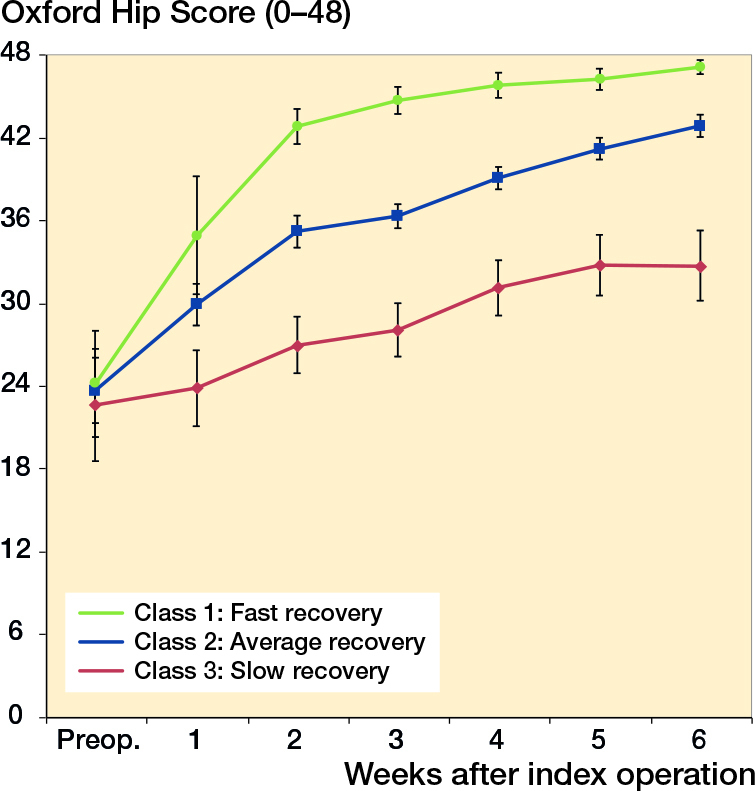
Trajectories of 6-week recovery after THA in a 3-class model (mean OHS [95% CI]).

## Supplementary Material

Supplemental Material

## References

[CIT0001] Adie S, Dao A, Harris I A, Naylor J M, Mittal R Satisfaction with joint replacement in public versus private hospitals: a cohort study. ANZ J Surg 2012; 82(9): 616–24. 22834486 10.1111/j.1445-2197.2012.06113.x

[CIT0002] Arthur H M, Blanchard C, Gunn E, Kodis J, Walker S, Toner B Exercise trajectories of women from entry to a 6-month cardiac rehabilitation program to one year after discharge. Biomed Res Int 2013; 2013: 121030. 24151580 10.1155/2013/121030 PMC3787588

[CIT0003] Baert I A C, Lluch E, Mulder T, Nijs J, Noten S, Meeus M Does pre-surgical central modulation of pain influence outcome after total knee replacement? A systematic review. Osteoarthr Cartilage 2016; 24(2): 213–23. 10.1016/j.joca.2015.09.002 26382109

[CIT0004] Bergés I M, Ottenbacher K J, Smith P M, Smith D Perceived pain and satisfaction with medical rehabilitation after hospital discharge. Clin Rehabil 2006; 20(8): 724–30. 16944829 10.1191/0269215506cre1006oa PMC1804256

[CIT0005] Chou C P, Yang D, Pentz M A, Hser Y I Piecewise growth curve modeling approach for longitudinal prevention study. Comput Stat Data An 2004; 46(2): 213–25.

[CIT0006] Crow R, Gage H, Hampson S, Hart J, Kimber A, Thomas H The role of expectancies in the placebo effect and their use in the delivery of health care: a systematic review. Health Technol Assess 1999; 3(3): 1–96. 10448203

[CIT0007] Dowsey M M, Liew D, Stoney J D, Choong P F M The impact of obesity on weight change and outcomes at 12 months in patients undergoing total hip arthroplasty. Med J Australia 2010; 193(1): 17–21. 20618108 10.5694/j.1326-5377.2010.tb03734.x

[CIT0008] Duncan T E, Duncan S C An introduction to latent growth curve modeling. Behav Ther 2004; 35(2): 333–63.

[CIT0009] Espehaug B, Havelin L I, Engesaeter L B, Langeland N, Vollset S E Patient satisfaction and function after primary and revision total hip replacement. Clin Orthop Relat Res 1998; (351): 135–48. 9646756

[CIT0010] Gandhi R, Davey J R, Mahomed N Patient expectations predict greater pain relief with joint arthroplasty. J Arthroplasty 2009; 24(5): 716–21. 18701241 10.1016/j.arth.2008.05.016

[CIT0011] Hahn S R, Kroenke K, Spitzer R L, Brody D, Williams J B W, Linzer M, deGruy F V The difficult patient. J Gen Intern Med 1996; 11(1): 1–8. 8691281 10.1007/BF02603477

[CIT0012] Hoeksma J B, Kelderman H On growth curves and mixture models. Infant Child Dev 2006; 15(6): 627–34.

[CIT0013] Jackson J L, Kroenke K Difficult patient encounters in the ambulatory clinic: clinical predictors and outcomes. Arch Intern Med 1999; 159(10): 1069–75. 10335683 10.1001/archinte.159.10.1069

[CIT0014] Judge A, Cooper C, Arden N K, Williams S, Hobbs N, Dixon D, Günther K-P, Dreinhoefer K, Dieppe P A Pre-operative expectation predicts 12-month post-operative outcome among patients undergoing primary total hip replacement in European orthopaedic centres. Osteoarthr Cartilage 2011; 19(6): 659–67. 10.1016/j.joca.2011.03.009 21447395

[CIT0015] Keurentjes J C, Fiocco M, So-Osman C, Onstenk R, Koopman-Van Gemert A W M M, Pöll R G, Kroon H M, Vliet Vlieland T P M, Nelissen R G Patients with severe radiographic osteoarthritis have a better prognosis in physical functioning after hip and knee replacement: a cohort-study. Baradaran HR, editor. PLoS One 2013; 8(4): e59500. 23573200 10.1371/journal.pone.0059500 PMC3616074

[CIT0016] Klapwijk L C M, Mathijssen N M C, Van Egmond J C, Verbeek B M, Vehmeijer S B W The first 6 weeks of recovery after primary total hip arthroplasty with fast track. Acta Orthop 2017; 88(2): 140–4. 28079428 10.1080/17453674.2016.1274865 PMC5385107

[CIT0017] Lewis G N, Rice D A, McNair P J, Kluger M Predictors of persistent pain after total knee arthroplasty: a systematic review and meta-analysis. Br J Anaesth 2015; 114(4): 551–61. 25542191 10.1093/bja/aeu441

[CIT0018] Nagin D S, Odgers C L Group-based trajectory modeling in clinical research. Annu Rev Clin Psychol 2010; 6(1): 109–38. 20192788 10.1146/annurev.clinpsy.121208.131413

[CIT0019] Nylund K L, Asparouhov T, Muthén B O Deciding on the number of classes in latent class analysis and growth mixture modeling: a Monte Carlo simulation study. Struct Equ Modeling 2007; 14(4): 535–69.

[CIT0020] Palazzo C, Jourdan C, Descamps S, Nizard R, Hamadouche M, Anract P, Boisgard S, Galvin M, Ravaud P, Poiraudeau S Determinants of satisfaction 1 year after total hip arthroplasty: the role of expectations fulfilment. BMC Musculoskelet Disord 2014; 15(1): 53. 24564856 10.1186/1471-2474-15-53 PMC3936828

[CIT0021] Sayers A, Wylde V, Lenguerrand E, Beswick AD, Gooberman-Hill R, Pyke M, Dieppe P, Blom AW Rest pain and movement-evoked pain as unique constructs in hip and knee replacements. Arthritis Care Res 2016; 68(2): 237–45. 10.1002/acr.22656 PMC5053254 26212349

[CIT0022] Smoot B, Cooper BA, Conley Y, Kober K, Levine JD, Mastick J, Topp K, Miaskowski C Differences in limb volume trajectories after breast cancer treatment. J Cancer Surviv 2016; 10(4): 772–82. 26678895 10.1007/s11764-015-0507-2 PMC4912957

[CIT0023] Tristaino V, Lantieri F, Tornago S, Gramazio M, Carriere E, Camera A Effectiveness of psychological support in patients undergoing primary total hip or knee arthroplasty: a controlled cohort study. J Orthop Trauma 2016; 17(2): 137–47. 10.1007/s10195-015-0368-5 PMC4882293 26220315

[CIT0024] Vissers M M, Bussmann J B, Verhaar J A N, Busschbach J J V, Bierma-Zeinstra S M A, Reijman M Psychological factors affecting the outcome of total hip and knee arthroplasty: a systematic review. Semin Arthritis Rheum 2012; 41(4): 576–88. 22035624 10.1016/j.semarthrit.2011.07.003

[CIT0025] Zou K, Wong J, Abdullah N, Chen X, Smith T, Doherty M, Zhang W Examination of overall treatment effect and the proportion attributable to contextual effect in osteoarthritis: meta-analysis of randomised controlled trials. Ann Rheum Dis 2016; 75: 1964–70. 26882927 10.1136/annrheumdis-2015-208387 PMC5099197

