# Supplementary material for: Early recovery trajectories after fast-track primary total hip arthroplasty: the role of patient characteristics
Source: Acta Orthop. 2018 Oct 23;89(6):597–602. doi: 10.1080/17453674.2018.1519095 (PMC6300723; doi:10.1080/17453674.2018.1519095)
Supplement: Supplemental Material [file IORT_A_1519095_SM3292.pdf]

Supplementary data

Table 2. Piecewise latent class growth model results

Number of classes		Intercept [a] (SE)	Slopes [b] (SE)	Number of free parameters	BIC [c]	BLRT [d]	Entropy [e]	Patients per class
1-class	Class 1	24 (0.76)	5.41 (0.47)<br>1.72 (0.15)	10	4291.627	–	–	94
2-class	Class 1	22 (1.2)	3.94 (0.61)<br>1.91 (0.23)	21	4024.293	p < 0.001	0.92	49/45
	Class 2	25 (1.0)	7.09 (0.74)<br>1.54 (0.17)					
3-class	Class 1	25 (1.9)	9.52 (0.91)<br>0.90 (0.12)	32	3912.545	p < 0.001	0.96	24/17/53
	Class 2	24 (0.89)	5.30 (0.46)<br>2.03 (0.15)					
	Class 3	22 (1.6)	2.68 (0.89)<br>1.73 (0.39)					
4-class	Class 1	25 (0.96)	5.84 (0.51)<br>1.93 (0.20)	43	3867.885	p < 0.001	0.94	26/33/18/17
	Class 2	24 (1.3)	4.54 (0.75)<br>1.86 (0.21)					
	Class 3	20 (1.7)	2.57 (0.84)<br>1.92 (0.52)					
	Class 4	25 (1.9)	9.56 (0.94)<br>0.89 (0.13)					

[a] Estimated average OHS at preoperative baseline.
[b] The first number refers to the estimated weekly growth rate in the first 2 weeks of the 6-week recovery period; the second number refers to the estimated weekly growth rate in the last 4 weeks of the 6-week recovery period.
[c] Bayesian Information Criterion, a lower value indicates better model fit.
[d] Bootstrapped Likelihood Ratio Test, tests whether a significant improvement in model fit occurred with the addition of an extra class.
[e] Entropy index (0–1), higher values indicate better overall accuracy of class separation.
